# A multi-drug resistant *Salmonella* Typhimurium ST213 human-invasive strain (33676) containing the *bla*_CMY-2_ gene on an IncF plasmid is attenuated for virulence in BALB/c mice

**DOI:** 10.1186/s12866-016-0633-7

**Published:** 2016-02-09

**Authors:** Magdalena Wiesner, Juan J. Calva, Víctor H. Bustamante, Deyanira Pérez-Morales, Marcos Fernández-Mora, Edmundo Calva, Claudia Silva

**Affiliations:** Departamento de Microbiología Molecular, Instituto de Biotecnología, Universidad Nacional Autónoma de México, Cuernavaca, Morelos Mexico; Department of Infectious Diseases, Instituto Nacional de Ciencias Médicas y Nutrición “Salvador Zubirán”, México City, Mexico; Present address: Grupo de Microbiología, Dirección de Investigación en Salud Pública, Instituto Nacional de Salud, Bogotá, Colombia

**Keywords:** Bacterial pathogenesis, Antimicrobial resistance, Multilocus sequence typing MLST, *Salmonella* virulence plasmid pSTV, Bacterial conjugation, IncA/C plasmid, IncF plasmid, ColE1-like plasmid, Plasmid co-integration, *Salmonella* pathogenicity islands SPIs

## Abstract

**Background:**

Classical strains of *Salmonella enterica* serovar Typhimurium (Typhimurium) predominantly cause a self-limiting diarrheal illness in humans and a systemic disease in mice. In this study, we report the characterization of a strain isolated from a blood-culture taken from a 15-year old woman suffering from invasive severe salmonellosis, refractory to conventional therapy with extended-spectrum cephalosporin (ESC).

**Results:**

The strain, named 33676, was characterized as multidrug-resistant *Salmonella* serogroup A by biochemical, antimicrobial and serological tests. Multilocus sequence typing (MLST) and XbaI macrorestrictions (PFGE) showed that strain 33676 belonged to the Typhimurium ST213 genotype, previously described for other Mexican Typhimurium strains. PCR analyses revealed the presence of IncA/C, IncFIIA and ColE1-like plasmids and the absence of the *Salmonella* virulence plasmid (pSTV). Conjugation assays showed that the ESC-resistance gene *bla*_CMY-2_ was carried on the conjugative IncF plasmid, instead of the IncA/C plasmid, as found in previously studied ST213 strains. Although the IncA/C plasmid conferred most of the observed antimicrobial resistances it was not self-conjugative; it was rather able to conjugate by co-integrating with the IncF plasmid. Strain 33676 was fully attenuated for virulence in BALB/c mice infections. Both type-three secretion system (T3SS), encoded in *Salmonella* pathogenicity islands 1 and 2 (SPI-1 and SPI-2), were functional in the 33676 strain and, interestingly, this strain produced the H2 FljB flagellin instead of the H1 FliC flagellin commonly expressed by *S. enterica* strains.

**Conclusions:**

Strain 33676 showed two main features that differentiate it from the originally described ST213 strains: 1) the *bla*_CMY-2_ gene was not carried on the IncA/C plasmid, but on a conjugative IncF plasmid, which may open a new route of dissemination for this ESC-resistance gene, and 2) it expresses the H2 FljB flagella, in contrast with the other ST213 and most Typhimurium reference strains. To our knowledge this is the first report of an IncF *bla*_CMY-2_-carrying plasmid in *Salmonella*.

**Electronic supplementary material:**

The online version of this article (doi:10.1186/s12866-016-0633-7) contains supplementary material, which is available to authorized users.

## Background

*Salmonella enterica* are Gram-negative flagellated bacteria that cause salmonellosis, a food-borne disease in humans causing gastroenteritis and bacteremia worldwide. The most prevalent *Salmonella* serovars isolated from humans and other animals are Typhimurium and Enteritidis [1, 2]. Typhimurium strains cause gastroenteritis in humans and several animals, whereas in mice they produce a systemic infection similar to the enteric fever generated by the human-restricted serovar Typhi [3, 4]. In most *S. enterica* serovars, the antigenic formula is composed by two flagellar phases (H1 and H2). That is, *Salmonella* can express two different flagellin subunits, FliC and FljB, from two divergently oriented genes. The expression of FliC and FljB is controlled by phase variation, which involves the inversion of a 1 kb DNA containing the promoter of *fljB*, to express either *fljB* or *fliC* genes [5]. Commonly, the *S. enterica* strains express the flagellar H1 phase. Although the role of flagella in motility, chemotaxis and pathogenesis is recognized, the biological significance of phase variation in *Salmonella* has been scarcely addressed [3, 6].

The majority of *Salmonella* virulence genes are clustered in regions distributed over the chromosome called *Salmonella* pathogenicity islands (SPIs), whereas others are found on the *Salmonella* virulence plasmid (pSTV) [7–10]. More than twenty SPIs have been described for *Salmonella* [11]; however, the well-studied are SPI-1 and SPI-2. Both SPI-1 and SPI-2 contain around 40 genes that encode a type-three secretion system (T3SS), which mediate the translocation of effector proteins into the eukaryotic host cells. The T3SS and effector proteins encoded in SPI-1 are necessary for *Salmonella* invasion of intestinal epithelial cells, and thus for the intestinal colonization leading to enteritis; whereas the T3SS and effector proteins encoded in SPI-2 are mainly required for *Salmonella* survival and replication inside macrophages, and hence for the systemic disease [3, 8]. The pSTV plasmid belongs to the F incompatibility group (IncF), harboring the *spv* operon, which is necessary to confer full virulence in mice [12–14]; however, the role of this plasmid in human infections is less clear. It has been accepted that pSTV is not required to cause gastroenteritis, but its association with human bacteremia is still in debate [12, 15, 16].

Antimicrobial resistance among *Salmonella* has been increasing, particularly in serovar Typhimurium [1]. Antimicrobial therapy is not required for most *Salmonella* infections, but it may be lifesaving in patients with or at risk for extra-intestinal infection [17]. Several studies have addressed the increased risk for hospitalization, invasive illness, and death posed by multi-drug resistant (MDR) *Salmonella* [18–20].

In Mexico, the results of an integrated food chain surveillance program showed that Typhimurium was the serovar most frequently associated with human infections, and particularly high MDR levels were recorded in both humans and food-animals [21]. Zaidi et al. reported the emergence of MDR Typhimurium strains with extended-spectrum cephalosporin (ESC) resistance in Yucatán, as the cause of severe enteric and systemic infections in children [22]. The ESC-resistance was found to be determined by the *bla*_CMY-2_ gene, which confers resistance to ceftriaxone (CRO), the antibiotic of choice for treating severe gastroenteritis and systemic infections. In a previous study, we analyzed a sample of 114 Typhimurium strains from the surveillance program in Mexico by multi-locus sequence typing (MLST), macro-restrictions (PFGE), and PCR typing for accessory genes involved in virulence and anti-microbial resistance [23]. We found that all the MDR strains with the ESC-resistant phenotype carried the *bla*_CMY-2_gene on a large plasmid (150 kb) of the IncA/C incompatibility group (IncA/C), and that these strains belonged to a new chromosomal multi-locus genotype, designated in the MLST database as sequence type 213 (ST213) [23, 24]. Another distinctive feature of the ST213 genotype was the lack of the pSTV, which contains virulence determinants and is characteristic of serovar Typhimurium, which was nevertheless present in strains with other genotypes in the Mexican population, such as ST19, ST302 and ST429 [23].

In this work we studied a *Salmonella* strain, named 33676, isolated from a blood-culture taken from a 15-year old young woman suffering a febrile illness with invasive severe pancolitis, refractory to conventional therapy with ESC. The aim of the study was to characterize genetic and phenotypic attributes of strain 33676, to get insights on the biology behind the unusual severe clinical manifestations observed in a patient with no signs of malnutrition, immunodeficiency or other underlying medical conditions. A battery of molecular and phenotypic approaches were applied to analyze strain 33676, including MLST, PFGE, PCR detection of plasmid markers, along with antimicrobial susceptibility tests, protein secretion profiles, and assays for virulence in the murine host. The usefulness of these molecular and phenotypic typing methods in the detection and classification of *Salmonella* strains, and in the context of host adaptation and the current epidemiological situation is discussed.

## Methods

### Ethics statement

Since this is a retrospective case-report of a patient cared during routine clinical practice on May 2011, no written or informed consent was obtained. Laboratory studies were part of the routine diagnostic work-out in a patient with diarrhea and a fever. There was no submission to an institutional review board (ethics committee) at the hospital, as this report did not imply the implementation and conductance of a designed research protocol with human participants. Blood and stool samples were collected by laboratory technicians of the hospital, as part of the routine clinical care of the patient. The unit of study in the present communication was a bacterial strain, isolated from this patient, which was characterized in a research laboratory. Microbiologists carrying out this task coded this bacterial strain omitting any disclosure of the patient’s identity.

The studies performed with animals were carried out in strict accordance with the recommendations in the guide for the Care and Use of Laboratory Animals of the National Institutes of Health, USA. The protocol was approved by the Committee on the Ethics of Animal Experiments of the Instituto de Biotecnología de la UNAM, Mexico (Permit Number: 286). Animals were sacrificed before signs of suffering appeared.

### Strain isolation, antimicrobial susceptibility tests, and serological classification

The bacterial strain described in this report was isolated from a previously healthy 15-year old young woman who attended the emergency room of a private hospital in Mexico City, with a 4-day duration clinical picture characterized by fever, vomiting, severe abdominal pain and watery bloody diarrhea. The patient was started with amoxicillin/clavulanic acid medication 72 h before hospital admission. Upon clinical examination, one gram of intravenous ceftriaxone every 12 h was commenced. At day 4, due to the adverse clinical course of the disease, and based on the report of the antibiotic susceptibility tests for the bacterium (*Salmonella*) isolated from stool and blood cultures (see below), ceftriaxone was changed to ertapenem. Clinical improvement 24 h after this therapeutic modification was observed. At day 9, the patient was discharged from the hospital with complete remission of all systemic and abdominal signs and symptoms.

The microbiological investigation of stool and blood samples was performed at the Instituto Nacional de Ciencias Médicas y de la Nutrición Salvador Zubirán, using the VITEK-2 AST-115 panel (bioMerieux), which allows microbial identification of bacteria and antibiotic susceptibility by the broth microdilution method according to Clinical and Laboratory Standards Institute [25].

The biochemical tests showed that both samples contained *Salmonella*. The serogroup determination was performed with *Salmonella* O antisera (Difco), used in slide agglutination tests for the identification of *Salmonella* by somatic (O) antigens. The flagellar antigen H was not assessed. For further studies, the strain isolated from the blood-culture was named as 33676 (Table 1).

**Table 1:** Bacterial strains used in this study

Strain	Features	Reference
33676	Typhimurium human invasive ST213 strain	This study
YU39	Typhimurium human invasive ST213 strain	[31]
SL1344	Typhimurium ST19 reference strain	[62]
Δ*ssrB*	SL1344 derivative, Δ*ssrB::kan*, strain MJW112	[63]
ΔSPI-1	SL1344 derivative, ΔSPI-1*::kan*	[64]
ΔSPI-2	SL1344 derivative, ΔSPI-2*::kan*	[65]
DH5α	*E. coli* laboratory strain	[26]

### DNA extraction, MLST and PFGE

Genomic DNA was extracted using standard laboratory protocols [26]. PCR amplifications were performed using Taq DNA polymerase (Invitrogen, Brazil), and the products were purified with a purification kit from Qiagen (Valencia, California, USA), and submitted for sequencing at Macrogen (Seoul, South Korea).

The seven loci used in the *S. enterica* MLST scheme were *aroC*, *dnaN*, *hemD*, *hisD*, *purE*, *sucA* and *thrA* [27]. The PCR primers and conditions used were those reported in the MLST web site (http://mlst.warwick.ac.uk/mlst/dbs/Senterica). The sequences at each locus were submitted to the web database for allele assignment, and the combination of alleles was assigned to a sequence type (ST).

The restriction of genomic DNA with XbaI was resolved by pulsed-field gel electrophoresis (PFGE) using the standardized protocol from PulsNet CDC [28]. The XbaI restriction patterns were clustered using the unweighted pair-group method with arithmetic averages. The analysis was performed with GelComparII using band matching and Dice coefficients with a 1.5 % band position tolerance.

### PCR detection of plasmids, virulence and antimicrobial-resistance genes

We determined by PCR the presence of plasmids of the incompatibility groups IncA/C, ColE1-like, IncFIIA, IncHI2, IncI1 and IncX, which are among the most frequently reported to carry antimicrobial resistance in enteric bacteria [29]. Three regions previously used as markers for the IncA/C plasmids were amplified: *bla*_CMY-2_, conferring resistance to ESC; *floR*, conferring resistance to chloramphenicol; and the 2 kb class 1 integron (*dfrA12*, *orfF* and *aadA2*), conferring resistance to trimethoprim and streptomycin [23, 24]. Three regions distributed along the pSTV were detected as well by PCR: *rck*, *spvC* and *traT*, involved in resistance to serum and survival in macrophages [12, 13, 30]. To detect the presence of the genes encoding flagellar subunits FliC and FljB, PCR amplification were performed for *fliC* and *fljB* genes. The PCR conditions were those previously reported [23, 24], and the primers used are listed in Additional file 1: Table S1. Primers used in this study.

DNA plasmid extraction was performed by the alkaline lysis procedure as reported elsewhere [23, 26], and plasmids were visualized by electrophoresis in 0.7 % agarose gels subjected to 60 V for 8 h.

### Conjugation and transformation experiments

To determine the transfer capabilities of the plasmid carrying the *bla*_CMY-2_ gene, conjugation experiments were performed as previously reported [24, 31]. A spontaneous rifampicin-resistant mutant of the nalidixic acid-resistant *Escherichia coli* DH5α strain was used as recipient (Table 1), and transconjugants were selected on rifampicin (100 μg/ml), nalidixic acid (60 μg/ml) and ceftriaxone (CRO) (2 μg/ml). Transfer frequencies were calculated as the number of transconjugants per donor. The conjugation experiments were repeated three times.

To obtain transformants with the different plasmids of strain 33676, the alkaline lysis plasmid extraction was used as donor DNA to electroporate competent DH5α strains. Ceftriaxone (2 μg/ml), tetracycline (12 μg/ml) and chloramphenicol (60 μg/ml), separately, were used for selection of transformants. Transformant colonies were analyzed by PCR with primers for the IncFIIA and IncA/C incompatibility groups (Additional file 1: Table S1. Primers used in this study) as described above.

### Competitive index (CI) assays for mice virulence

Mice infections and calculation of the competitive index (CI) were performed as described previously [32, 33]. Briefly, five BALB/c mice were infected orally with an equal mix of 10^4^ bacteria of SL1344 standard reference strain and test strains. The test strains were the two Mexican Typhimurium strains isolated from human invasive infections, 33676 or YU39, and the Δ*ssrB::kan* SL1344 derivative strain lacking the SsrB regulator necessary for the expression of SPI-2 genes (Table 1). After three days of infection, animals were sacrificed by CO_2_ asphyxiation followed by cervical dislocation, and the bacterial load in the spleen and liver was quantified from organ homogenates by plating serial 10-fold dilutions of cell lysates on LB agar plates and counting the colony-forming units (CFUs) for each strain. Ampicillin resistance of 33676 and YU39, as well as kanamycin resistance of Δ*ssrB*, were used to differentiate these strains from SL1344, which is not resistant to these antibiotics. The CI was calculated as the ratio 33676/SL1344, YU39/SL1344 or Δ*ssrB*/SL1344 of CFUs obtained from the mouse organs, divided by their respective ratio from the initial inoculum [33].

### Protein secretion analysis and flagella characterization

Analysis of the proteins secreted by the type III secretion systems encoded in SPI-1 and SPI-2 was performed as described previously [34]. Secretion mediated by SPI-1 and SPI-2 was determined in bacterial cultures grown for 9 h in LB and for 16 h in N-minimal medium (N-MM) at pH 5.8, respectively. Proteins contained in 1.5 ml samples of culture supernatants were precipitated by the addition of 10 % (v/v) trichloroacetic acid and overnight incubation at 4 °C. Precipitated proteins were concentrated by centrifugation at 16,000 *g* for 30 min at 4 °C. Pellets were resuspended in SDS-PAGE loading buffer containing 10 % saturated Tris base. Samples were subjected to SDS-PAGE analysis, using 12 % polyacrylamide gels. The SPI-1-encoded proteins SipA, SipB, SipC and SipD, as well as the flagellar protein FliC or FljB, were detected by staining with Coomassie Brilliant Blue R-250. The SPI-2-encoded SseB protein was detected by Western blot with an anti-SseB polyclonal antibody, as described previously [34]. The intense band of about 57 kDa secreted by strain 33676 was excised from the gel, digested with trypsin, and analyzed by liquid chromatography-mass spectrometry at the Unidad de Proteómica of the Instituto de Biotecnología, UNAM.

## Results

### Strain 33676 was identified as an MDR *Salmonella* serogroup A 1, 4, 5 (+)

We studied a strain isolated from a patient with severe pancolitis and bacteremia. Bacterial isolates were obtained both from stool and blood samples. The biochemical tests showed that the isolates belonged to *Salmonella*. Serological classification showed that the isolates were from the *Salmonella* serogroup A, factor 1, 4, 5 (+). The isolates were resistant by minimum inhibitory concentrations (MICs) break-points to 12 out of 19 antimicrobials: ampicillin (MIC > =32), amoxicillin/clavulanic acid (MIC > =32), ticarcillin/clavulanic acid (MIC > =128), piperacillin/tazobactam (MIC > =128), cefazolin (MIC > =64), ceftazidime (MIC > =64), ceftriaxone (MIC > =64), amikacin (MIC < =2), gentamicin (MIC > =16), ciprofloxacin (MIC =2), nitrofurantoin (MIC = 128) and trimethoprim/sulfamethoxazole (MIC > =320). Sensitivity was recorded to cefepime, ertapenem, imipenem, meropenem, norfloxacin, tigecycline and fosfomycine. Resistance to chloramphenicol (30 μg/ml), streptomycin (100 μg/ml) and tetracycline (12 μg/ml) was also recorded by growth in LB broth supplemented with these antibiotics, which were later used to analyze the conjugation ability of the strain (see below).

For further studies, the strain isolated from the blood-culture was named as 33676. The antimicrobial susceptibility tests showed that strain 33676 was MDR, which explains the empiric therapeutic failure obtained when the patient was treated with amoxicillin/clavulanic acid or ceftriaxone, whereas positive response was obtained with ertapenem, and the patients’ health was restored.

### MLST and PFGE showed that strain 33676 belongs to Typhimurium ST213

The combination of alleles for the seven loci used in the *S. enterica* MLST scheme showed that strain 33676 belongs to ST213, which was initially reported for Mexican Typhimuirum strains [23], and has been recently reported for other Typhimurium strains from the United Kingdom and Netherlands (http://mlst.warwick.ac.uk/mlst/dbs/Senterica). Achtman et al. had provided evidence that MLST can be used to assign strains to *Salmonella* serovars, since the housekeeping genes used in the MLST scheme are conserved among salmonellae and hence reflect the evolutionary history of the strains [35]. To set strain 33676 in the context of our previously analyzed Typhimurium ST213 strains, sub-typing was performed by restriction of genomic DNA with XbaI resolved by pulsed-field gel electrophoresis (PFGE). The comparison of the XbaI restriction profile with those of the Mexican Typhimurium strains reported by Wiesner et al. [23] showed that strain 33676 grouped within the ST213 cluster (Additional file 2: Figure S1. Strain 33676 belongs to the Mexican ST213 population). Under the evidence provided by the MLST and PFGE data, strain 33676 belongs to the Typhimurium ST213 evolutionary lineage.

### Strain 33676 carries IncA/C, IncF and ColE-1-like plasmids

**Fig. 1:**
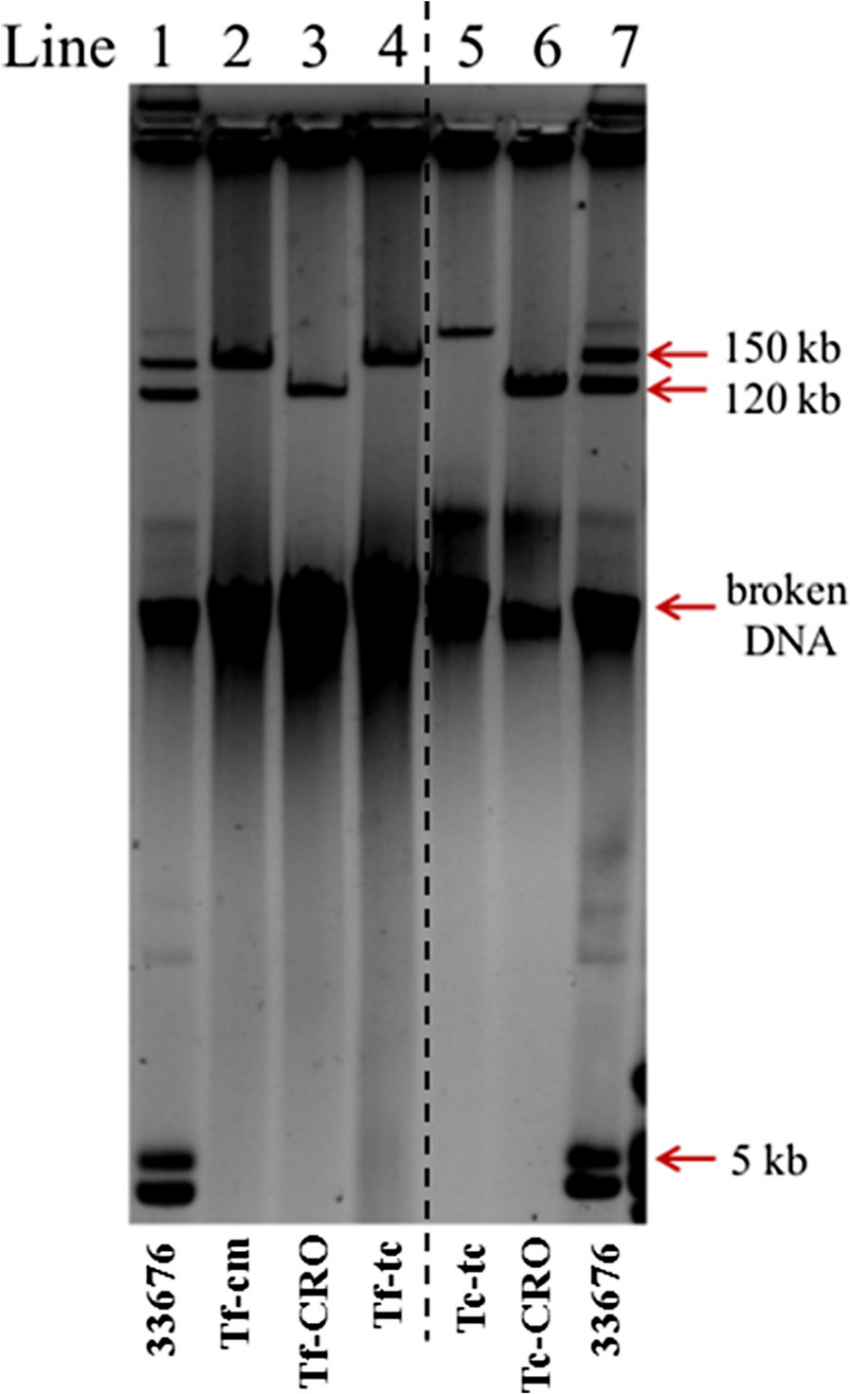
Plasmid profiles for strain 33676 and its transformant or transconjugant derivatives. Strain 33676 carries three plasmids of about 150, 120 and 5 kb, corresponding to IncA/C, IncF and ColE1-like plasmids, respectively (lanes 1 and 7). *E.coli* DH5α derivatives were obtained by conjugation or transformation. Transformants were obtained with chloramphenicol (Tf-cm, lane 2), ceftriaxone (Tf-CRO, lane 3), and tetracycline (Tf-tc, lane 4); transconjugants were obtained with tetracycline (Tc-tc, lane 5), and ceftriaxone (Tc-CRO, lane 6)

The PCR screening showed that strain 33676 harbored plasmids of incompatibility groups IncA/C, IncFIIA and ColE1-like. The amplification of the other three IncA/C plasmid markers, the antibiotic resistance genes *bla*_CMY-2_ and *floR*, and the 2 kb integron, were also positive, suggesting the presence of an IncA/C plasmid carrying the *bla*_CMY-2_ gene. PCR amplifications for three markers distinctive of the pSTV (*rck*, *spvC* and *traT*) were negative, indicating that 33676 does not carry a pSTV. However, the positive amplification with the IncFIIA primers indicated that an IncF plasmid, other than pSTV, was present in 33676. The plasmid profile of strain 33676 showed the presence of three plasmids of about 150, 120 and 5 kb (Fig. 1, lanes 1 and 7), which corresponded to the IncA/C, IncF, and ColE1-like plasmids, respectively (see below).

### The blaCMY-2 gene is borne on a conjugative IncF plasmid

The mobility of the 33676 *bla*_CMY-2_ gene was studied by conjugation assays selecting DH5α transconjugants resistant to ceftriaxone. DH5α transconjugants resistant to CRO were found at a frequency of 5.9-1.9 x 10^−6^. The transconjugants were evaluated by PCR for the presence of the repA/C, *bla*_CMY-2_ and *floR* markers of the IncA/C plasmid. Unexpectedly, the transconjugants were positive only for *bla*_CMY-2_, but not for repA/C and *floR* markers, indicating that *bla*_CMY-2_ was not carried on the IncA/C plasmid. Positive amplification was recorded with the IncFIIA primers, indicating that in strain 33676 the *bla*_CMY-2_ gene was located on a conjugative IncF plasmid. The plasmid profile displayed by the transconjugants revealed that the 120 kb plasmid was the IncF plasmid carrying the *bla*_CMY-2_ gene (Fig. 1, lane 6).

### The MDR IncA/C plasmid is not conjugative but can co-integrate with the conjugative IncF plasmid

A transformation assay was performed, using the alkaline-lysis plasmid extract of 33676 as donor DNA, and DH5α strain as recipient. Selection was performed with ceftriaxone to obtain the IncF plasmid with the *bla*_CMY-2_ gene, and with chloramphenicol or tetracycline to select for the IncA/C plasmid. The plasmid profiles and the PCR typing with the IncFIIA and IncA/C primers, confirmed that the transformant colonies obtained with ceftriaxone only carried the IncF plasmid (Fig. 1, lane 3), while the colonies obtained with chloramphenicol or tetracycline only contained the IncA/C plasmid (Fig. 1, lanes 2 and 4).

The amplification products of the *bla*_CMY-2_ gene present in the IncF plasmid transformants, and the 2 kb integron (conferring resistance to streptomycin and trimethoprim/sulfamethoxazole) present in the IncA/C plasmid transformants, were sequenced and showed nucleotide sequences identical to those reported for the other Mexican ST213 strains [23]. Antimicrobial susceptibility tests for the transformant colonies showed that the IncF plasmid accounted for resistance to penicillin and cephalosporin antibiotics, whereas the IncA/C plasmid provided resistance to the other antibiotic classes (chloramphenicol, gentamicin, streptomycin, tetracycline and trimethoprim/sulfamethoxazole). These results indicated that most of the resistance determinants were carried by the MDR IncA/C plasmid, while the IncF plasmid carried only the *bla*_CMY-2_ gene which accounts for penicillin and cephalosporin resistances.

To determine if the IncA/C plasmid was conjugative, a conjugation experiment was performed selecting with tetracycline, using strain 33676 as the donor and DH5α as the recipient strain. Transconjugant colonies were obtained at a frequency of 1 x 10^−5^. Transconjugants were positive for the amplification with the IncA/C primers; however, they were also positive for amplification with the IncF primers, indicating that both plasmids were transferred. The plasmid profiles of the tetracycline-resistant transconjugants displayed only one plasmid, larger than the IncA/C plasmid (Fig. 1, lane 5). PCR amplifications with the *bla*_CMY-2_, *floR* and integron regions were positive, and the antimicrobial resistance profiles showed resistance to 10 out of the 15 resistances, as found for the parental 33676 strain, suggesting that the large plasmid was formed by the co-integration of the complete IncA/C and IncF plasmids.

We hypothesized that the IncA/C plasmid of strain 33676 was not self-conjugative, and that co-integration with the IncF plasmid was necessary to be transferred. To test this hypothesis, a conjugation assay was performed, using as donor a DH5α transformant strain carrying the IncA/C plasmid, and DH5α as recipient. No transconjugants were obtained, supporting the notion that the IncA/C plasmid is not self-conjugative, as previously found for the other ST213 strains [24, 31].

### Strains 33676 and YU39 are fully attenuated for virulence in BALB/c mice

Classical Typhimurium strains, such as SL1344, cause severe systemic infections in mice [36]. To determine whether strain 33676 displayed a similar or different virulence phenotype in mice as compared to that of classical strains, the ability of this invasive strain to compete with the reference strain SL1344 in colonizing the liver and spleen of mice was tested by CI assays. For these assays, another ST213 strain was included, YU39, which was isolated in 2005 in Yucatán, Mexico, from the blood-culture of an eight-year old boy with hepatomegaly and severe thrombocytopenia with hemorrhagic syndrome. This strain was also referred to as YUHS 05-78 in previous studies (Table 1) [23, 24]. Due to the fact that SsrB is a transcriptional regulator required for the expression of the *Salmonella* pathogenicity island 2 genes, which are essential for the intracellular proliferation of *Salmonella* and thus for its systemic infection in mice [3], a Δ*ssrB* strain derivative of SL1344 was used as a negative control in these assays. Interestingly, after three days of infection, strains 33676 and YU39 were not detected or isolated from the mice organs at very low numbers, a similar phenomenon observed for the mutant Δ*ssrB* strain, whereas a high number of bacteria was detected for the wild-type strain SL1344. Therefore, extremely low CIs values were obtained for the ratios 33676/SL1344, YU39/SL1344 and, as expected, for Δ*ssrB*/SL1344 (Table 2). These results indicate that strains 33676 and YU39 are fully attenuated for systemic infection in mice.

**Table 2:** Competitive indexes (CIs)^a^ for mice infections between the reference strain SL1344, human invasive strains 33676 and YU39, and the SPI-2 mutant strain Δ*ssrB*

Ratio	Spleen	Liver
33676/SL1344	<5 x 10^−8^	< 7 x 10^−8^
YU39/SL1344	< 5 x 10^−8^	< 8 x 10^−7^
Δ*ssrB* /SL1344	< 8 x 10^−8^	< 3 x 10^−8^

^a^ CI values lower than 1 indicate virulence attenuation. For most mice no bacteria were detected for strains 33676, YU39 and Δ*ssrB*; thus, the values showed represent the lower detection limit for these assays

### Strain 3376 has functional SPI-1- and SPI-2-encoded type III secretion systems and produces the flagellar subunit FljB

**Fig. 2:**
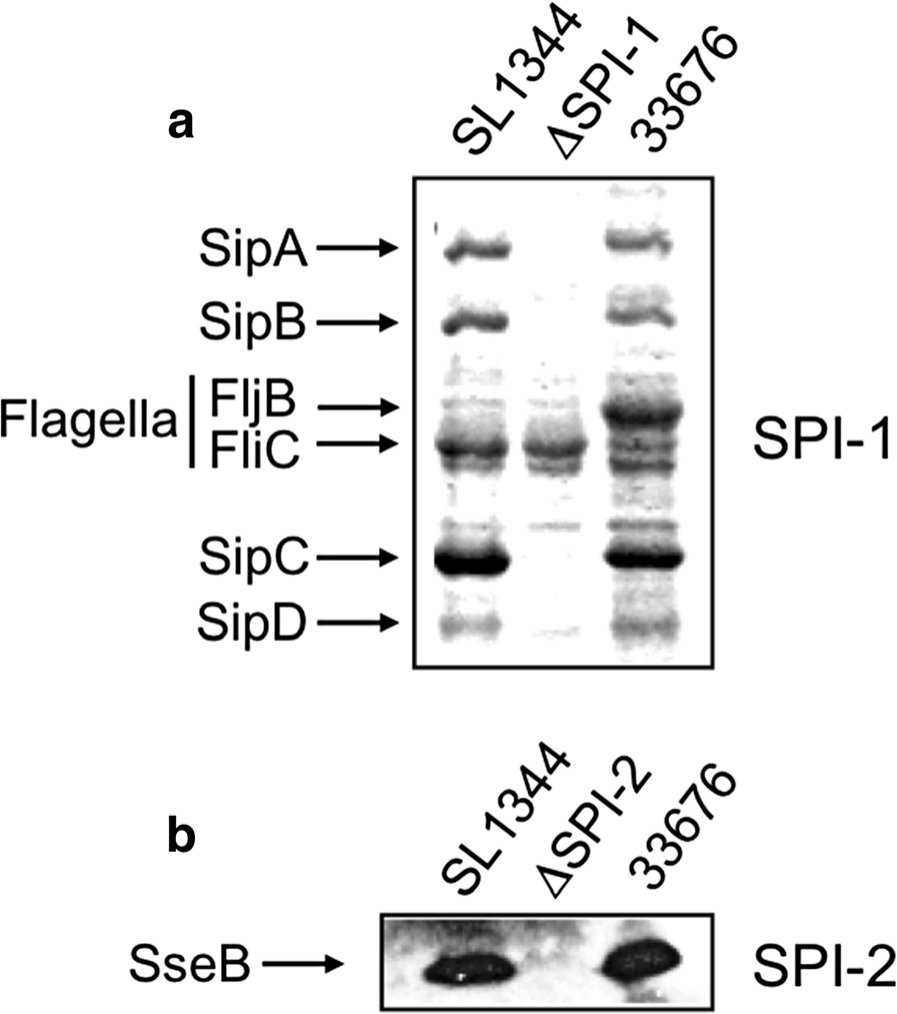
Strain 33676 has functional SPI-1 and SPI-2-encoded T3SSs and secretes FljB flagellin. Analysis of secreted proteins from culture supernatants was performed for strains SL1344, 33676 and ΔSPI-1 or ΔSPI-2 grown in LB (**a**) or N-MM (**b**). TCA-precipitated proteins SipA, SipB, SipC and SipD, secreted through the T3SS encoded in SPI-1 (**a**), and protein SseB, secreted through the T3SS encoded in SPI-2 (**b**), were detected by Coomassie blue staining and by Western blot, respectively. FliC and FljB (**a**) are flagellin subunits proteins whose secretion is independent of SPI-1

SPI-1 and SPI-2 are essential for virulence; each one encodes a T3SS through which *Salmonella* translocates effector proteins from the bacteria into the cytoplasm of host cell [3, 4]. In order to determine the functionality of these T3SSs, protein secretion mediated by SPI-1 and SPI-2 was analyzed for strain 33676, as well as for the reference strain SL1344 and for ΔSPI-1 and ΔSPI-2 mutants derived from SL1344, which lack SPI-1 and SPI-2, respectively (Table 1). Both strains 33676 and SL1344 showed the typical SPI-1-mediated protein secretion profile (SipA, SipB, SipC and SipD proteins) and the secreted SPI-2-encoded protein SseB. As expected, these SPI-1 or SPI-2 encoded proteins were not detected in the respective ΔSPI-1 or ΔSPI-2 mutants (Fig. 2a and b). Typhimurium can present phase variation to alternatively express two different flagellin subunit proteins, FliC and FljB [37], which can be detected in the protein secretion analysis for SPI-1. Whereas strain SL1344 showed FliC, strain 33676 presented a higher molecular weight protein of about 57 kDa (Fig. 2a); which was identified by protein sequence analysis as the flagellin subunit FljB, indicating that strain 33676 expresses phase H2 flagella. PCR amplifications for the *fliC* and *fljB* genes from strain 33676 were positive, and product sequencing showed that both genes were identical to those of other Typhimurium strains, such as DT104 (HF937208), indicating that the differential expression of *fljB* was not due to the absence a *fliC* gene.. Therefore, these results showed that both T3SSs are functional in strain 33676, and revealed that strain 33676 produces the flagellar subunit FljB in the growth conditions tested, instead the FliC phase that is common for most *S. enterica* strains.

## Discussion

The results of MLST along with PFGE provided unequivocal evidence that this strain belonged to the ST213 genotype of serovar Typhimurium (Additional file 2: Figure S1. Strain 33676 belongs to the Mexican ST213 population). PCR typing, in combination with plasmid profiling, allowed to discover that in strain 33676 the ESC-resistance gene, *bla*_CMY-2_, was carried on an IncF plasmid. This is in contrast with the previously analyzed ST213 strains, which carried this gene on IncA/C plasmids, as found for other Typhimurium strains [24, 38]. Despite the fact that strain 33676 indeed harbors an IncA/C plasmid, which accounts for most of the MDR phenotype, the *bla*_CMY-2_ gene maps to a conjugative IncF plasmid. Although IncF plasmids have been involved in antibiotic resistance in enteric bacteria [39–41], there is only one report of an IncF plasmid carrying *bla*_CMY-2_ in *E. coli* [42]; however, to our knowledge this is the first report of an IncF *bla*_CMY-2_-carrying plasmid in *Salmonella*.

In a previous conjugation study, with another ST213 donor strain (YU39), we found that the *bla*_CMY-2_ gene was transferred to recipient strains by means of co-integration with a conjugative IncX1 plasmid, or by transposition of the *bla*_CMY-2_ region into the conjugative IncX1 plasmid [31]. In the present study, when strain 33676 was challenged for conjugation using as selection an antibiotic carried by the IncA/C plasmid (tetracycline), chimeric plasmids containing the molecular markers and antibiotic resistance profile of both IncA/C and IncF plasmids were obtained. The co-integration of the MDR IncA/C plasmid with the conjugative IncF-*bla*_CMY-2_ carrying plasmids in strain 33676, resembles the co-integration found among the IncA/C-*bla*_CMY-2_ plasmid and the conjugative IncX1 plasmid previously recorded in strain YU39 [31]. In both cases, the non-conjugative IncA/C plasmids took advantage of the conjugation machinery of a co-resident plasmid to transfer their antimicrobial resistance capabilities. In fact, it is possible to observe a faint band of the size of the co-integrate formed by the IncF and IncA/C on the plasmid profile of strain 33676 (Fig. 1, lanes 1 and 7), but not in the IncA/C and IncF transformants or transconjugant strains, supporting the notion that the co-integrate plasmid is naturally formed at low frequencies in the wild-type strain, even under non-selective pressure conditions without antibiotic. Taken together, these phenomena highlight the plastic nature of the plasmid compartments of the genomes of these ST213 Typhimurium strains.

In contrast to the classical Typhimurium strains, such as SL1344, which cause gastroenteritis in humans and systemic infection in mice, the Typhimurium ST213 strains 33676 and YU39 were isolated from human systemic infections and both were fully attenuated for virulence in mice (Table 2). A common feature of the ST213 strains is the lack of pSTV [23]. Many studies have demonstrated that pSTV is necessary for full virulence in the mouse [12, 13]. Evidence indicates that, in mice, the *spv* genes enable Typhimurium strains to infect the spleen and the liver by increasing the rate of bacterial replication within host cells. Plasmid-cured strains are able to colonize and persist in the spleen and the liver, but bacterial growth is controlled by host defenses and infection does not develop [14]. This is in agreement with the findings of Kapperud et al. [43], which studied a large Typhimurium outbreak traced to contaminated chocolate, and found that the strains lacking the pSTV were not virulent to mice, but caused acute, hemorrhagic diarrhea in children less than two years old [43]. Furthermore, we have tested other ST213 strains (lacking pSTV) and none of them were virulent in mice (data not shown). Therefore, the lack of pSTV likely explains the attenuated virulence of ST213 strains in mice. However, chromosomal-encoded factors are also required and can determinate differential virulence phenotype in mice of Typhimurium strains [44]. García-Quintanilla et al. demonstrated that the transfer of the pSTV from mice-virulent strains (SL1344 and ATCC 14028) did not provide virulence in mice to the non-virulent strain LT2 cured from its pSTV [45]. Furthermore, Heithoff et al. [46] suggested that *Salmonella* isolates derived from human salmonellosis patients are distinct from those of animal (chicken, cow, dog, horse, sheep and turkey) origin. They found that a fraction (19 %) of the Typhimurium strains (harboring the pSTV) derived from human salmonellosis patients were virulent in mice, in contrast with the virulent phenotype exhibited by all the animal isolates (harboring pSTV) examined [46].

Previously, since we found that in the Mexican Typhimurium population the pSTV was significantly associated with human isolates, and that ST213 strains lacking pSTV caused most of the systemic infection cases and were significantly associated with animal sources, we suggested that pSTV could be involved in human host adaptation [23]. However, the involvement of pSTV in human systemic infections is still controversial and deserves more research [12, 15, 16, 47]. Together, these studies indicate that bacterial-host interactions are complex, and several genetic elements are involved in the outcome of infection, and, that virulence in mice cannot be directly extrapolated to human virulence and vice versa [3, 30, 43, 47].

In addition to the defect in virulence showed by ST213 strains in mice, our preliminary results support the notion that strains 33676 and YU39 have a diminished ability to invade, but not to replicate within HeLa cells (data not shown). The absence of pSTV could be related to the invasion defect showed by 33676 and YU39 strains, since it has been demonstrated that pSTV is also necessary for cell invasion [4, 12, 48, 49].

The mechanisms employed by the pSTV-devoid ST213 strains to cause systemic disease in humans open new avenues for the study of *Salmonella*-host interactions. In the present study, it was demonstrated that the T3SSs from SPI-1 and SPI-2, which are essential for *Salmonella* virulence, are functional in strain 33676 (Fig. 2). Furthermore, it was found that strain 33676 expresses FljB flagellar subunit (Fig. 2a), which is unusual in Typhimurium; most of the reference strains, such as SL1344 (Fig. 2a), LT2 and 14028S express FliC flagella [6, 37]. Flagella-based motility is required for the initial contact of Typhimurium with epithelial cells [50]. Even when strain 33676 expresses the FljB flagellar subunit, instead of FliC expressed by strains SL1344 and YU39, the motility of these three strains was similar on LB plates containing soft agar (data not shown), which supports that a difference in motility is not involved in the invasion defect showed by strains 33676 and YU39. In a previous study, the effect of FljB and FliC on mice virulence was determined, by constructing phase-locked derivative strains [6]. The authors found that a Typhimurium classical strain locked-ON (expressing only FljB) was attenuated for virulence in the mouse, and the authors concluded that the ability to switch to the FliC phase has a selective advantage for Typhimurium to survive in mice [6]. However, in this study we found that, although the 33676 and YU39 strains express FljB and FliC (Fig. 2a and Additional file 3: Figure S2), respectively, they show similar virulence phenotypes (Table 2). Therefore, the expression of FljB, instead FliC, probably does not determine the virulence attenuation of the 33676 strain in mice, but it could be relevant for infection to humans. It has been established that a decreased production of FliC flagella reduces the inflammatory response [51], but the effect of FljB flagella on the human immune response remains to be studied.

ST213 was reported to be an emergent genotype in Mexico, characterized by the lack of pSTV, and the presence of multiple-drug resistance determinants, most of which are carried by IncA/C plasmids [23, 24]. We alerted about the emergence of the MDR genotype ST213 in Mexico, carrying resistance to ESC, which caused six out of the seven human systemic infections cases included in the study [22, 23]. The finding that strain 33676 (isolated in 2011) belongs to the MDR ST213 lineage indicates that this genotype continues to be a leading cause of disease in Mexico.

Strain 33676 shared most of the features studied in the ST213 surveillance population. However, two distinct features distinguished the originally described ST213 strains from strain 33676. First, the *bla*_CMY-2_ gene was not carried on the IncA/C plasmid, but on a conjugative IncF plasmid. And second, strain 33676 expresses the H2 FljB flagella. The antimicrobial resistance profile of strain 33676 increased, since ciprofloxacin resistance was not recorded in the 2000-2005 isolates [23]. These findings indicate that the emergent ST213 genotype is spreading and diversifying in Mexico.

Strain 33676 caused invasive disease in a young woman with no signs of malnutrition, immunodeficiency or other underlying medical conditions, which is unusual; since *Salmonella* systemic infections are more likely to occur in young children, immunologically compromised patients, and patients with comorbid medical conditions, such as malnutrition [3, 17, 52]. Invasive MDR Typhimurium strains have emerged as prominent cause of bloodstream infections worldwide in the last three decades [18–20, 23, 53–56]. Recently, the Typhimurium genotype ST313 has emerged as a new pathogen in sub-Saharan Africa, [51, 52]. ST313 is an MDR genotype characterized by harboring a version of pSTV carrying antibiotic resistance genes, and by signs of chromosomal degradation, with a higher number of pseudogenes as compared with other Typhimurium strains [55]. The ST313 genome degradation could account for adaptation to cause invasive disease in human beings, and also human-to-human transmission [52]. These two features are similar to those of the human-restricted serovar Typhi. In this respect, preliminary analysis of the complete genome of strain 33676 [57] showed a four-fold higher number of pseudogenes than those found in the YU39 genome [58]. Further functional analyses are necessary to address the impact of the pseudogenes on the pathogenesis of strain 33676.

We agree with Davies and Davies [59], in that antibiotic resistance can be considered as a marker concurrent with virulence factors; as some MDR strains have also acquired increased virulence and enhanced transmissibility [59]. Since many studies have addressed the importance of the animal reservoirs in the spread of MDR *Salmonella* throughout the food chain [2, 21, 60, 61], it is necessary to make efforts to restart the epidemiological surveillance program in Mexico to address the prevalence of ST213 strains, and the emergence of new variants, in the human population and the animal-production settings.

## Conclusion

In this study, a bacterial isolate that caused severe gastrointestinal and systemic (bacteremia) infections in a healthy young woman was characterized using a battery of molecular tools. Two main features distinguished strain 33676 from the originally described ST213 strains. First, the *bla*_CMY-2_ gene was not carried on the IncA/C plasmid, but on a conjugative IncF plasmid, which may open a new route of dissemination for this ESC-resistance gene. Second, strain 33676 expresses the H2 FljB flagella, in contrast with the other ST213 and most Typhimurium reference strains. The expression of FljB, instead FliC, probably does not determine the virulence attenuation of the 33676 strain in mice, but it could be relevant for infection to humans.

To our knowledge this is the first report of an IncF *bla*_CMY-2_-carrying plasmid in *Salmonella*. Moreover, the co-integration of the MDR IncA/C plasmid with the conjugative IncF-*bla*_CMY-2_ carrying plasmid highlights the plastic nature of the plasmid compartment of this Typhimurium strain.

## Additional files

Additional file 1: Table S1. Primers used in this study. (PDF 17 kb) Additional file 2 Figure S1. Strain 33676 belongs to the Mexican ST213 population. The dendrogram depicts the genetic relationships among Mexican Typhimurium strains based on XbaI restrictions resolved by pulsed-field gel electrophoresis (PFGE). The PFGE fingerprints were clustered by the UPGMA algorithm using Dice coefficients with 1.5 % band position tolerance. The columns at the right side indicate the strain name, year of isolation, sequence type (ST), resistance to ceftriaxone (CRO) and the State of Mexico where the strains were isolated (YU, Yucatán; MI, Michoacán; SL, San Luis Potosí; SO, Sonora), according to the data obtained by Wiesner et al. (2009) [23]. The position of strain 33676, isolated in Mexico City (DF, Distrito Federal), is indicated by the red dotted-line rectangle. The blue solid-line divides the ST213 strains from the strains with other STs (ST19, ST302 and ST429). (PDF 2999 kb) Additional file 3: Figure S2. The strain YU39 has a functional SPI-1-encoded T3SS and secretes FliC flagellin. Analysis of secreted proteins from culture supernatants was performed for strains SL1344, ΔSPI-1 and YU39 grown in LB medium. TCA-precipitated proteins SipA, SipB, SipC and SipD, secreted through the T3SS encoded in SPI-1, were detected by Coomassie blue staining. FliC is a flagellin subunit protein whose secretion is independent of SPI-1. (PDF 118 kb)
