# Supplementary material for: A multi-drug resistant Salmonella Typhimurium ST213 human-invasive strain (33676) containing the blaCMY-2 gene on an IncF plasmid is attenuated for virulence in BALB/c mice
Source: BMC Microbiol. 2016 Feb 9;16:18. doi: 10.1186/s12866-016-0633-7 (PMC4748464; doi:10.1186/s12866-016-0633-7)
Supplement: Additional file 1: Table S1. — Primers used in this study. (PDF 17 kb) [file 12866_2016_633_MOESM1_ESM.pdf]

Table S1. Primers used in this study.

Primer	Region	Sequence	Size (bp)	Reference
Inc screening				
A/C-F	*IncA/C*	ACTGAATTCGCGAAACTGGGGAAATGTG	2,589	[1]
A/C-R		TGTGTCGACGGTTCGTTCGTTGCGTTTCA		[1]
mobA-F	*ColE1-like*	GATCAGTTTCGCACGTTCCAG	900	[2]
mobA-R		GGGAACTCATTGATGCCACG		
FIIA-F	*FIIA*	CCTTCACACGACGTTCCACT	900	This study
FIIA-R		CGCCAGGTAAAGAACCCGAA		This study
HI2-F	*IncHI2*	TTTCTCCTGAGTCACCTGTTAACAC	644	[3]
HI2-R		GGCTCACTACCGTTGTCATCCT		
I1-F	*IncI1*	CGAAAGCCGGACGGCAGAA	139	[3]
I1-R		TCGTCGTTCCGCCAAGTTCGT		
N-F	*IncN*	GTCTAACGAGCTTACCGAAG	559	[3]
N-R		GTTTCAACTCTGCCAAGTTC		
stbD5-F	*IncX1*	CTCATAAGCCCTCCGCTTGTCT	2,000	[2]
pir3-R		TTTCACTGCAAAACATTTCTTACGC		[2]
pA/C screening				
CMY-F	*bla*$_{CMY-2}$	ATAACCACCCAGTCACGC	600	[4]
CMY-F		CAGTAGCGAGACTGCGCA		[4]
CS-F	*integron*	GGCATCCAAGCAGCAAG	2,000	[5]
CS-R		AAGCAGACTTGACCTGA		[5]

floR-F	*floR*	CCGCGTGGGCCTATACGCTG	1,100	[1]
floR-R		GAGCCGAAGGAGCACCAGCC		[1]
pSTV screening				
SpvC-1	*spvC*	ACTCCTTGCACAACCAAATGCGGA	550	[4]
SpvC-2		TGTCTCTGCATTTCGCCATCA		[4]
traT-F	*traT*	GATGGTTACACTGGTCAG	500	[4]
traT-R		TCTGAGATCTGTACGTCG		[4]
rck-F	*rck*	TCGTTCTGTCCTCACTGC	500	[4]
rck-R		TCATAGCCCAGATCGATG		[4]
Flagella screening				
fliC-F	*fliC*	GTCTGGATACGCTGAATGTG	650	This study
fliC-R		GTGACCTTCGGCTTTACTTG		This study
fljB-F	*fljB*	GCCATATTTCAGCCTCTCGCCCG	600	This study
fljB-R		TGTCGATAACCTGGATGACACAGG		This study

References.

1. Wiesner M, Calva E, Fernandez-Mora M, Cevallos MA, Campos F, Zaidi MB et al. *Salmonella* Typhimurium ST213 is associated with two types of IncA/C plasmids carrying multiple resistance determinants. BMC Microbiol. 2011;11(1):9. doi:1471-2180-11-9 [pii]

10.1186/1471-2180-11-9.

2. Wiesner M, Fernández-Mora M, Cevallos MA, Zavala-Alvarado C, Zaidi MB, Calva E et al. Conjugative transfer of an IncA/C plasmid-borne *bla*$_{CMY-2}$ gene through genetic re-arrangements with an IncX1 plasmid. BMC Microbiol. 2013;13:264. doi:10.1186/1471-2180-13-264.

3. Carattoli A, Bertini A, Villa L, Falbo V, Hopkins KL, Threlfall EJ. Identification of plasmids by PCR-based replicon typing. J Microbiol Methods. 2005;63(3):219-28.

4. Wiesner M, Zaidi MB, Calva E, Fernandez-Mora M, Calva JJ, Silva C. Association of virulence plasmid and antibiotic resistance determinants with chromosomal multilocus genotypes in Mexican *Salmonella enterica* serovar Typhimurium strains. BMC Microbiol. 2009;9:131.

5. Levesque C, Piche L, Larose C, Roy PH. PCR mapping of integrons reveals several novel combinations of resistance genes. Antimicrob Agents Chemother. 1995;39(1):185-91.
