# Supplementary material for: A multi-drug resistant Salmonella Typhimurium ST213 human-invasive strain (33676) containing the blaCMY-2 gene on an IncF plasmid is attenuated for virulence in BALB/c mice
Source: BMC Microbiol. 2016 Feb 9;16:18. doi: 10.1186/s12866-016-0633-7 (PMC4748464; doi:10.1186/s12866-016-0633-7)
Supplement: Additional file 2 Figure S1. — Strain 33676 belongs to the Mexican ST213 population. The dendrogram depicts the genetic relationships among Mexican Typhimurium strains based on XbaI restrictions resolved by pulsed-field gel electrophoresis (PFGE). The PFGE fingerprints were clustered by the UPGMA algorithm using Dice coefficients with 1.5 % band position tolerance. The columns at the right side indicate the strain name, year of isolation, sequence type (ST), resistance to ceftriaxone (CRO) and the State of Mexico where the strains were isolated (YU, Yucatán; MI, Michoacán; SL, San Luis Potosí; SO, Sonora), according to the data obtained by Wiesner et al. (2009) [23]. The position of strain 33676, isolated in Mexico City (DF, Distrito Federal), is indicated by the red dotted-line rectangle. The blue solid-line divides the ST213 strains from the strains with other STs (ST19, ST302 and ST429). (PDF 2999 kb) [file 12866_2016_633_MOESM2_ESM.pdf]

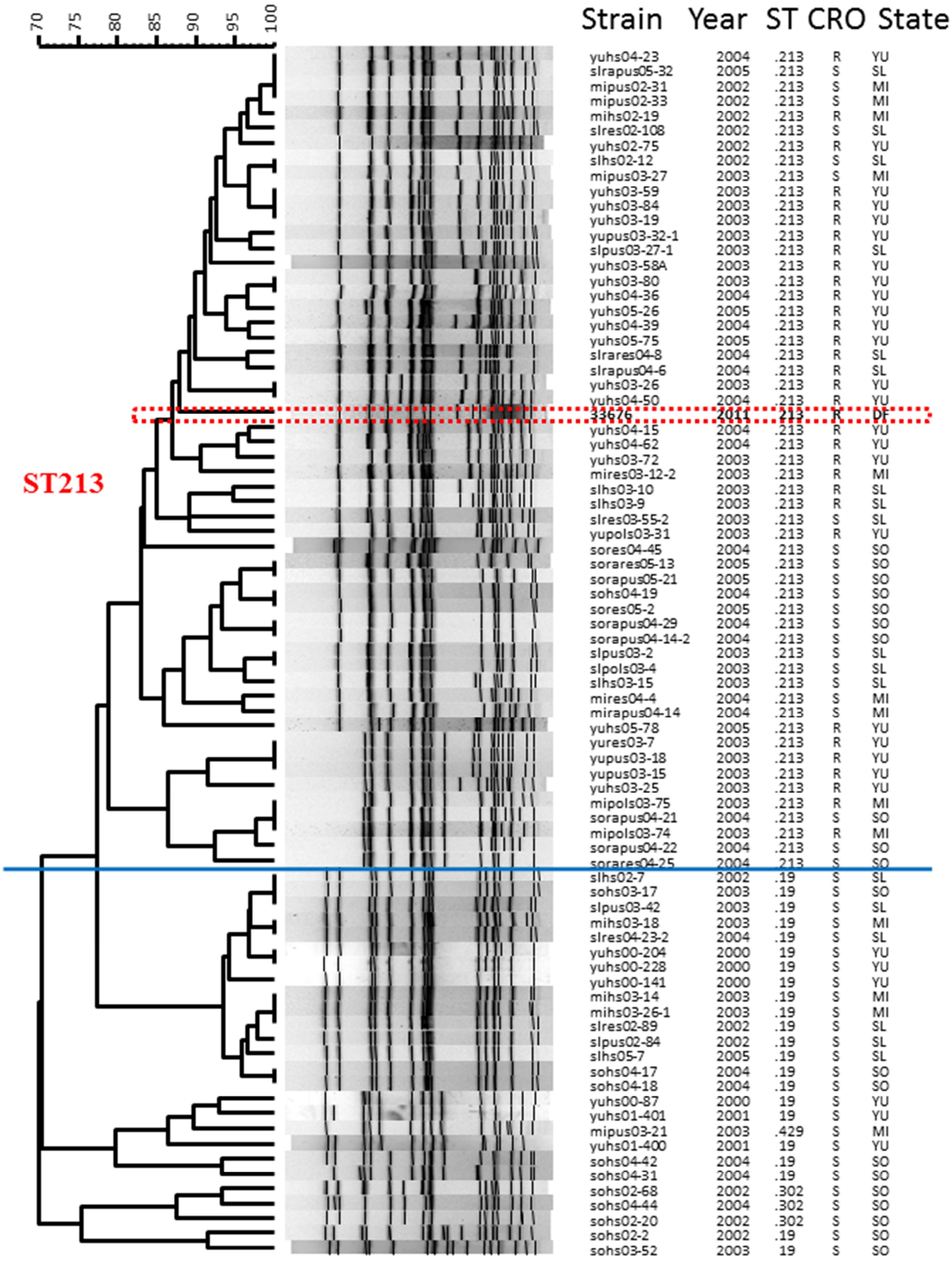

Strain	Year	ST	CRO	State
yuhs04-23	2004	.213	R	YU
slrapus05-32	2005	.213	S	SL
mipus02-31	2002	.213	S	MI
mipus02-33	2002	.213	S	MI
mihs02-19	2002	.213	R	MI
slres02-108	2002	.213	S	SL
yuhs02-75	2002	.213	R	YU
slhs02-12	2002	.213	S	SL
mipus03-27	2003	.213	S	MI
yuhs03-59	2003	.213	R	YU
yuhs03-84	2003	.213	R	YU
yuhs03-19	2003	.213	R	YU
yupus03-32-1	2003	.213	R	YU
slpus03-27-1	2003	.213	R	SL
yuhs03-58A	2003	213	R	YU
yuhs03-80	2003	.213	R	YU
yuhs04-36	2004	.213	R	YU
yuhs05-26	2005	.213	R	YU
yuhs04-39	2004	.213	R	YU
yuhs05-75	2005	.213	R	YU
slrares04-8	2004	.213	R	SL
slrapus04-6	2004	.213	R	SL
yuhs03-26	2003	.213	R	YU
yuhs04-50	2004	.213	R	YU
33676	2011	213	R	DF
yuhs04-15	2004	.213	R	YU
yuhs04-62	2004	.213	R	YU
yuhs03-72	2003	.213	R	YU
mires03-12-2	2003	.213	R	MI
slhs03-10	2003	.213	R	SL
slhs03-9	2003	.213	R	SL
slres03-55-2	2003	.213	S	SL
yupols03-31	2003	.213	R	YU
sores04-45	2004	213	S	SO
sorares05-13	2005	.213	S	SO
sorapus05-21	2005	.213	S	SO
sohs04-19	2004	.213	S	SO
sores05-2	2005	.213	S	SO
sorapus04-29	2004	.213	S	SO
sorapus04-14-2	2004	.213	S	SO
slpus03-2	2003	.213	S	SL
slpols03-4	2003	.213	S	SL
slhs03-15	2003	.213	S	SL
mires04-4	2004	.213	S	MI
mirapus04-14	2004	.213	S	MI
yuhs05-78	2005	.213	R	YU
yures03-7	2003	.213	R	YU
yupus03-18	2003	.213	R	YU
yupus03-15	2003	.213	R	YU
yuhs03-25	2003	.213	R	YU
mipols03-75	2003	.213	R	MI
sorapus04-21	2004	.213	S	SO
mipols03-74	2003	.213	R	MI
sorapus04-22	2004	.213	S	SO
sorares04-25	2004	.213	S	SO
slhs02-7	2002	.19	S	SL
sohs03-17	2003	.19	S	SO
slpus03-42	2003	.19	S	SL
mihs03-18	2003	.19	S	MI
slres04-23-2	2004	.19	S	SL
yuhs00-204	2000	19	S	YU
yuhs00-228	2000	19	S	YU
yuhs00-141	2000	19	S	YU
mihs03-14	2003	.19	S	MI
mihs03-26-1	2003	.19	S	MI
slres02-89	2002	.19	S	SL
slpus02-84	2002	.19	S	SL
slhs05-7	2005	.19	S	SL
sohs04-17	2004	.19	S	SO
sohs04-18	2004	.19	S	SO
yuhs00-87	2000	19	S	YU
yuhs01-401	2001	19	S	YU
mipus03-21	2003	.429	S	MI
yuhs01-400	2001	19	S	YU
sohs04-42	2004	.19	S	SO
sohs04-31	2004	.19	S	SO
sohs02-68	2002	.302	S	SO
sohs04-44	2004	.302	S	SO
sohs02-20	2002	.302	S	SO
sohs02-2	2002	.19	S	SO
sohs03-52	2003	19	S	SO

Figure S1. Strain 33676 belongs to the Mexican ST213 population. The dendrogram depicts the genetic relationships among Mexican Typhimurium strains based on XbaI restrictions resolved by pulsed-field gel electrophoresis (PFGE). The PFGE fingerprints were clustered by the UPGMA algorithm using Dice coefficients with 1.5% band position tolerance. The columns at the right side indicate the strain name, year of isolation, sequence type (ST), resistance to ceftriaxone (CRO) and the State of Mexico where the strains were isolated (YU, Yucatán; MI, Michoacán; SL, San Luis Potosí; SO, Sonora), according to the data obtained by Wiesner et al. (2009) [1]. The position of strain 33676, isolated in Mexico City (DF, Distrito Federal), is indicated by the red dotted-line rectangle. The blue solid-line divides the ST213 strains from the strains with other STs (ST19, ST302 and ST429).

Reference.

1. Wiesner M, Zaidi MB, Calva E, Fernandez-Mora M, Calva JJ, et al. (2009) Association of virulence plasmid and antibiotic resistance determinants with chromosomal multilocus genotypes in Mexican *Salmonella enterica* serovar Typhimurium strains. BMC Microbiol 9: 131.
