# Supplementary material for: A multi-drug resistant Salmonella Typhimurium ST213 human-invasive strain (33676) containing the blaCMY-2 gene on an IncF plasmid is attenuated for virulence in BALB/c mice
Source: BMC Microbiol. 2016 Feb 9;16:18. doi: 10.1186/s12866-016-0633-7 (PMC4748464; doi:10.1186/s12866-016-0633-7)
Supplement: Additional file 3: Figure S2. — The strain YU39 has a functional SPI-1-encoded T3SS and secretes FliC flagellin. Analysis of secreted proteins from culture supernatants was performed for strains SL1344, ΔSPI-1 and YU39 grown in LB medium. TCA-precipitated proteins SipA, SipB, SipC and SipD, secreted through the T3SS encoded in SPI-1, were detected by Coomassie blue staining. FliC is a flagellin subunit protein whose secretion is independent of SPI-1. (PDF 118 kb) [file 12866_2016_633_MOESM3_ESM.pdf]

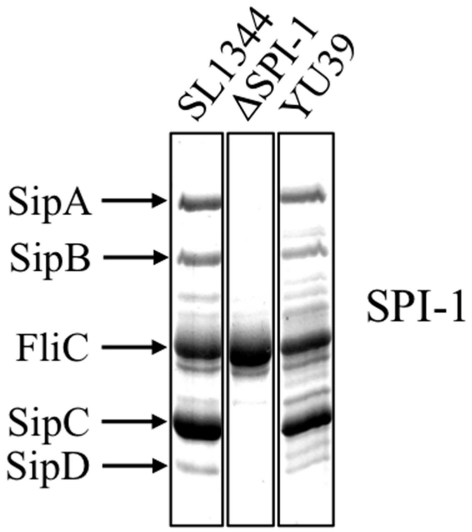

Figure S2. The strain YU39 has a functional SPI-1-encoded T3SS and secretes FliC flagellin. Analysis of secreted proteins from culture supernatants was performed for strains SL1344, ΔSPI-1 and YU39 grown in LB medium. TCA-precipitated proteins SipA, SipB, SipC and SipD, secreted through the T3SS encoded in SPI-1, were detected by Coomassie blue staining. FliC is a flagellin subunit protein whose secretion is independent of SPI-1.
